# Risk prediction models for central venous catheter-related complications in children: a systematic review

**DOI:** 10.1186/s12887-026-06919-y

**Published:** 2026-04-27

**Authors:** Ruijuan Liu, Lu Li, Yansheng Peng, Liping Liao, Wenyu Huang, Yuan Li

**Affiliations:** 1 Nursing Department, Affiliated Hospital of Zunyi Medical University, No. 149, Dalian Road, Huichuan District, Zunyi, Guizhou 563003 P. R. China; 2 Nursing College, Zunyi Medical University, Zunyi, Guizhou 563003 P. R. China

**Keywords:** Central Venous Catheter, Children, Risk Prediction Model, CLABSI, CRT, Systematic Review

## Abstract

**Background:**

Central venous catheters (CVCs) are widely used in pediatric care; however, their indwelling procedure can lead to severe complications such as catheter-associated infections and thrombosis. These complications significantly increase the suffering of pediatric patients, prolong hospitalization, and pose life-threatening risks. Although multiple risk prediction models have been developed to identify high-risk pediatric patients, a systematic and comprehensive evaluation of these models is still lacking.

**Objective:**

We systematically reviewed published risk prediction models for central venous catheter-related complications ( CLABSI and CRT ) in children, both domestically and internationally. The review assessed their predictive performance, validation status, and methodological quality, aiming to provide a basis for the selection of appropriate models in clinical practice.

**Methods:**

Following the PRISMA guidelines, a systematic search was conducted in databases including PubMed, Web of Science, Embase, Cochrane Library, CINAHL, CNKI, Wanfang, VIP, and CBM for relevant literature from their inception to December 31, 2025. Two researchers independently screened the literature, extracted data, and assessed the risk of bias and applicability using the PROBAST tool. A descriptive analysis was performed on the model construction methods, performance metrics, and validation status.

**Results:**

A total of 15 studies were included in this analysis. Among them, three studies developed prediction models for CLABSI, all constructed using logistic regression(LR), with modeling AUCs ranging from 0.793 to 0.812; only one model underwent external validation. Twelve studies developed prediction models for CRT, employing diverse modeling methods (such as LR and machine learning). The modeling AUCs ranged from 0.74 to 0.957, of which four models underwent external validation (AUC 0.745–0.922). The PROBAST assessment indicated that most studies had a high risk of bias, primarily due to issues such as single-center retrospective design, highly subjective predictive factors, and inconsistent outcome evaluation criteria. The applicability of the models was generally satisfactory.

**Conclusion:**

Existing risk prediction models for central venous catheter-related complications in children demonstrate potential in terms of discrimination. However, models specifically for CLABSI are limited in number and insufficiently validated. Both types of models commonly exhibit methodological flaws such as a high risk of bias. Currently, these models primarily serve as exploratory tools for risk screening and cannot yet be used as standalone evidence for clinical decision-making. Future efforts should focus on conducting multicenter prospective studies, standardizing diagnostic criteria and model development/validation procedures, and promoting the integration of models into clinical systems to enhance their reliability and clinical applicability.

**Supplementary Information:**

The online version contains supplementary material available at 10.1186/s12887-026-06919-y.

## Introduction

Central Venous Catheters (CVCs), which primarily include Peripherally Inserted Central Catheters (PICC), tunneled or non-tunneled central venous catheters, and Totally Implantable Venous Access Ports (PORT) [1], serve as an indispensable lifeline in modern pediatric medical care. They are widely used for fluid resuscitation, long-term intravenous therapy, parenteral nutrition, blood purification, and hemodynamic monitoring in critically ill children [2]. However, the prolonged indwelling of CVCs carries risks of various complications [3]. These complications not only significantly increase patient suffering, prolong hospital stays, and impose a greater economic burden on families [4, 5], but are also important contributors to increased morbidity and mortality [6].

Central Line-Associated Bloodstream Infection (CLABSI) and Central Venous Catheter-Related Venous Thrombosis (CRT) are the most common and severe complications associated with central venous catheters. Catheter-related infections represent a major category of hospital-acquired infections. A study in China reported a CLABSI incidence of 0.6 per 1,000 catheter-days in pediatric hematology-oncology patients [7]. A study from South Korea documented an incidence of 0.93 per 1,000 catheter-days in children receiving long-term CVCs [8]. Another study conducted in the Pediatric Intensive Care Unit (PICU) showed a CLABSI incidence of 12.5 per 1,000 catheter-days [9]. In high-risk populations, such as neonates, the incidence was reported as 5.6 per 1,000 catheter-days [10], while studies from Neonatal Intensive Care Units (NICUs) in developed European countries reported rates ranging from 1.8 to 8.4 per 1,000 catheter-days [11]. CLABSI can lead to severe consequences, including sepsis and septic shock [12]. Research data indicate that approximately 17% of pediatric patients with CLABSI develop severe sepsis during the initial stage of infection, and 6% to 11% progress to severe systemic complications such as suppurative thrombophlebitis or infective endocarditis. Among immunocompromised children (e.g., those with malignancies or neutropenia) and those using implanted venous access devices, the incidence of severe complications can exceed 35% [13–15].

CRT may lead to post-thrombotic syndrome, characterized by vascular occlusion, limb swelling, and pain. In more severe cases, thrombus detachment can cause pulmonary embolism, posing a life-threatening risk [16, 17]. The incidence of CRT in critically ill patients reaches 16.9%, often resulting in partial or complete occlusion of the venous lumen. Approximately 0.9% of CRT patients may develop pulmonary embolism due to thrombus detachment [18]. Reported incidence rates of Catheter-Associated Deep Vein Thrombosis (CADVT) in children range from 14% to 28% [19, 20]. Additionally, although other complications such as catheter displacement, occlusion, and fracture occur frequently, their urgency and severity are generally secondary to infections and thrombosis [21]. Therefore, effectively preventing CLABSI and CRT has become a core indicator of pediatric intravenous therapy and nursing quality.

To identify high-risk pediatric patients and implement precise, proactive prevention strategies, clinical researchers are dedicated to developing various risk prediction models [22–24]. These models aim to quantify the probability of individual complications by integrating multiple risk factors, including demographic characteristics, underlying diseases, catheter features, and nursing practices. This approach assists clinical healthcare professionals in early identification of high-risk populations, optimizing catheter placement decisions, and enhancing targeted interventions [25, 26]. Ideally, a robust risk prediction model not only facilitates rapid screening of high-risk groups but also provides evidence-based support for developing individualized prevention and control plans [27]. This can help reduce the incidence of complications while avoiding potential risks associated with overtreatment.

As research in this field expands, multiple prediction models targeting different pediatric subgroups have been published. However, these models vary significantly in their development processes, validation quality, performance metrics, and applicability. To date, no study has systematically and comprehensively evaluated these models, making it difficult for clinical healthcare professionals to determine which model is more reliable and suitable for their specific patient populations. Additionally, the limitations and areas for improvement of existing models remain unclear.

This study aims to conduct a systematic review to comprehensively search and rigorously evaluate published risk prediction model studies on central venous catheter-related complications in children, both domestically and internationally. By synthesizing the predictive performance, validation status, and methodological quality of existing models, this review seeks to provide a scientific basis for selecting reliable and effective prediction tools in clinical practice. Furthermore, by summarizing the strengths and limitations of current models, it aims to offer constructive recommendations for developing more efficient and generalizable next-generation risk prediction models, ultimately advancing the safety management of central venous catheters in children toward greater precision and scientific rigor.

## Methods

### Study design and registration

This systematic review adhered to the recommendations outlined in the Preferred Reporting Items for Systematic Reviews and Meta-Analyses (PRISMA 2020) statement [28]. The protocol for this review has been registered with PROSPERO (Registration ID: CRD420251164446).

### Search strategy

This study searched a total of nine databases, including the Chinese databases CNKI, Wanfang Database, VIP Database, and CBM, and the English databases PubMed, Web of Science, Cochrane Library, Embase, and CINAHL. The search employed a combination of MeSH terms and free-text terms. Search terms included: “Child” / “Pediatric” / “Children” / “Central Venous Catheter” / “Peripherally Inserted Central Catheter” / “Totally Implantable Venous Access Port” / “CVC” / “PICC” / “Port” / “Complication” / “Adverse Effects” / “Blood-Borne Infect” / “Catheter-Related Infect” / “CRBSI” / “CLABSI” / “Bloodstream Infection” / “Blood Poison” / “Venous Thrombosis” / “Catheter-Related Thrombosis” / “Blood Clot” / “CRT” / “Risk Predict” / “Risk Score” / “Risk Assess” / “Score” / “Prognostic Model” / “Nomogram Model” / “Nomograms” / “Score Development” / “Risk Calculator” / “Machine Learning”. The initial search was conducted from inception to October 9, 2025. The search terms were expanded, and a secondary search was performed from inception to December 31, 2025. The search strategy for PubMed is presented in Table 1 as an example. The search strategy for this study was peer-reviewed by two evidence-based medicine experts to ensure comprehensiveness and accuracy. Please refer to the appendix for details.

**Table 1:** Search Strategy

#1	Child[Mesh Terms] OR Pediatrics[Mesh Terms] OR Children[Title/Abstract]
#2	Central Venous Catheters[Mesh Terms] OR “Central Venous Catheter*”[Title/Abstract] OR “Peripherally Inserted Central Catheter*”[Title/Abstract] OR“Totally Implantable Venous Access Port”[Title/Abstract] OR CVC [Title/Abstract] OR PICC[Title/Abstract] Or Port[Title/Abstract]
#3	Complication*[Title/Abstract] OR Adverse Effects[Title/Abstract] OR “Blood-Borne Infect*”[Title/Abstract] OR “Catheter-Related Infect*”[Title/Abstract] OR CRBSI[Title/Abstract] OR CLABSI[Title/Abstract] OR “Bloodstream Infect*”[Title/Abstract] OR Blood Poison*[Title/Abstract] OR Venous Thrombosis[Title/Abstract] OR “Catheter-Related Thrombosis”[Title/Abstract] OR “Blood Clot*”[Title/Abstract] Or CRT[Title/Abstract]
#4	Risk Predict*[Title/Abstract] OR Risk Score[Title/Abstract] OR Risk Assess*[Title/Abstract] OR Score[Title/Abstract] OR Risk Model[Title/Abstract] OR Nomogram* Model[Title/Abstract] OR Score Development[Title/Abstract] OR Risk Calculator[Title/Abstract] OR Machine Learning[Title/Abstract]
#5	#1 AND #2 AND #3 AND #4

### Inclusion and exclusion criteria 

#### Inclusion criteria

- ① Population: Children who had any type of central venous catheter inserted;
- ② Study design: Cross‑sectional, retrospective, or prospective studies;
- ③ Intervention/Model: Studies on the development and/or validation of risk prediction models for central venous catheter‑related complications in children;
- ④ Outcome: The primary outcomes were catheter‑related bloodstream infection and catheter‑related thrombosis;
- ⑤ Catheter type: All clinically common pediatric CVCs (PICC, tunneled/non‑tunneled CVCs, PORT) were included;
- ⑥ Setting: Both inpatient and outpatient settings; indications for catheterization included hemodialysis, long‑term intravenous therapy, parenteral nutrition, hemodynamic monitoring, etc.

#### Exclusion criteria

- ① Language: Publications in languages other than Chinese or English;
- ② Accessibility and Completeness: Studies for which the full text was unavailable or data were incomplete, including conference abstracts, etc.;
- ③ Number of Predictors: Models containing fewer than two predictor variables;
- ④ Population: Studies where the study population consisted solely of neonates or preterm infants.

### Core outcome definitions

CLABSI is a term used by the National Healthcare Safety Network of the Centers for Disease Control and Prevention (CDC). It refers to a primary bloodstream infection (not community-acquired and not related to an infection at another site) occurring in a patient with a central venous catheter in place or within 48 h after its removal [29]. The CDC defines CLABSI as having at least one positive blood culture result obtained from a peripheral vein, accompanied by clinical signs of infection and not related to an infection at another site [30].

CRT refers to venous thrombosis caused by catheter-related factors (such as vascular endothelial injury, blood flow stagnation, etc.), encompassing thrombosis in the main venous trunk, branches, and tributaries [31, 32]. CRT is a specific type of Venous Thromboembolism (VTE) associated with CVCs, accounting for approximately 50–80% of pediatric Deep Vein Thrombosis (DVT) cases [33, 34]. CADVT is a subtype of CRT [35], specifically referring to catheter-associated thrombosis occurring in the deep venous system. In this study, individual mentions of VTE, DVT, and CADVT are uniformly referred to as CRT.

### Literature screening and data extraction

The processes of literature screening and data extraction were strictly conducted according to PRISMA guidelines. Retrieved records were imported into EndNote for duplicate removal. Literature screening was initially performed by reviewing titles and abstracts against the inclusion and exclusion criteria. The full texts of potentially eligible studies were then retrieved and reviewed to determine final inclusion. A data extraction form was developed based on the Checklist for Critical Appraisal and Data Extraction for Systematic Reviews of Prediction Modelling Studies (CHARMS) [36] and general study information. Data extraction was independently performed by two researchers, followed by cross-checking. Any discrepancies were resolved through discussion or adjudication by a third researcher. The extracted data from the finally included studies included: first author, publication year, country, study population, study design, data source, predicted outcome, method for handling missing data, sample size for model development, modelling method, validation method, model performance, number of candidate variables, model presentation, number of predictors in the final model, and model reporting.

### Bias risk assessment of included studies

Two researchers trained in evidence-based practice independently assessed the risk of bias and applicability of the included studies using the Prediction Model Risk of Bias Assessment Tool (PROBAST) [37]. Their assessments were cross-checked, and any disagreements were resolved by a third researcher. The risk of bias assessment covered four domains: participants, predictors, outcome, and analysis, comprising a total of 20 questions. Each question was answered as “yes,” “no,” or “unclear.” A domain was rated as “low risk of bias” if all questions within it were answered “yes.” It was rated as “high risk of bias” if one or more questions were answered “no.” If any question was answered “unclear,” the domain was rated as “unclear risk of bias.” The overall risk of bias for a study was judged as “low” if all four domains were rated as low risk. It was judged as “high” if any domain was rated as high risk. If any domain was rated as unclear risk while the others were low risk, the overall judgment was “unclear risk of bias.”

In the PROBAST tool, “applicability” refers to the degree of alignment between the included prediction models and the objectives of this systematic review across three dimensions: target population, predictors, and outcome measures. Specifically, it evaluates the relevance of the model results to predicting the risks of CLABSI and CRT associated with pediatric CVCs, which is the focus of this study.

### Statistical analysis

A descriptive analysis was conducted on the development, performance and validation, risk of bias, and applicability assessment results of the included risk prediction models.

## Results

### Literature screening process and results

**Fig. 1:**
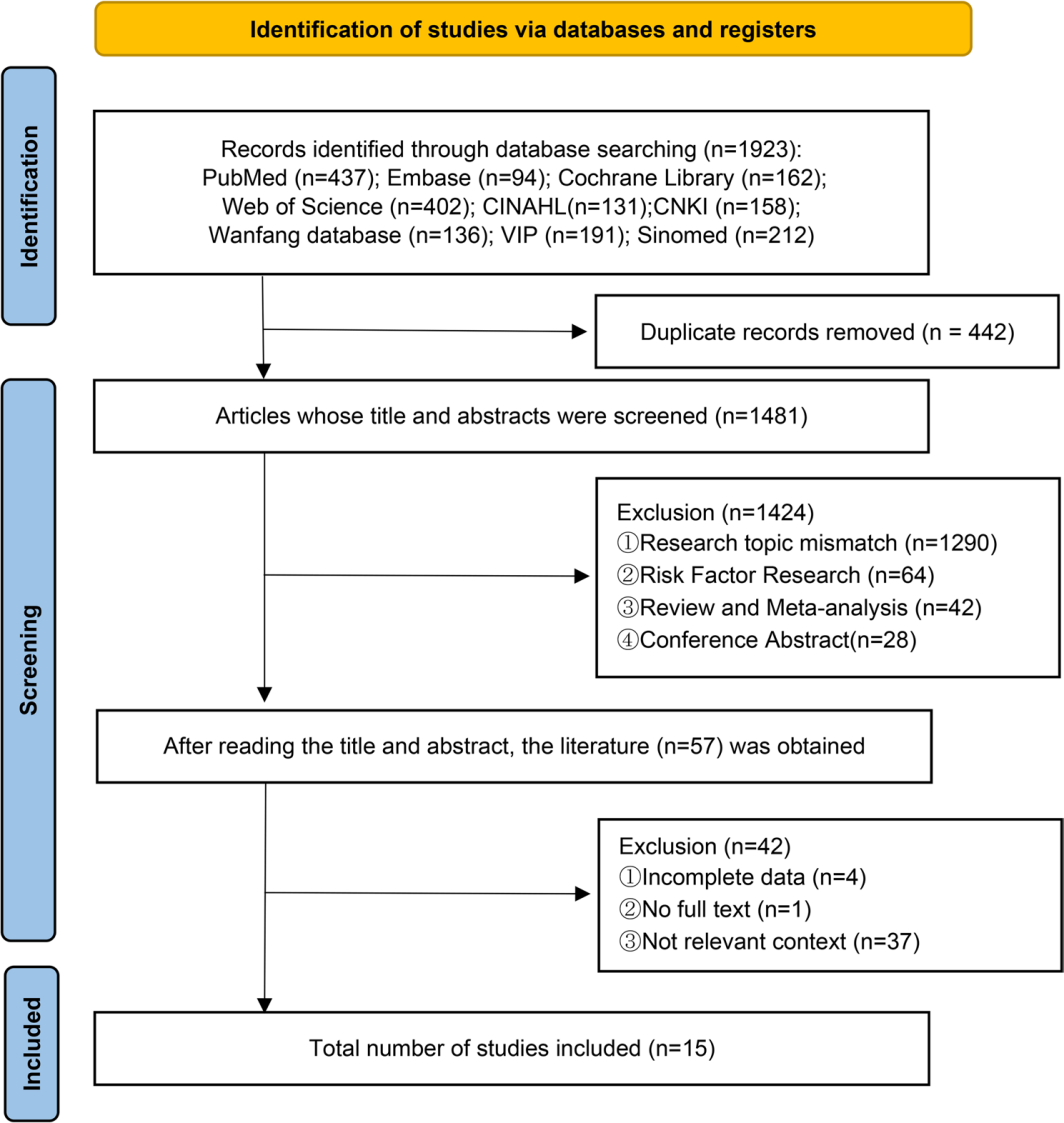
Flowchart of Literature Screening

A total of 1923 relevant articles were identified through initial screening. After a stepwise screening process, 15 studies were ultimately included [9, 19, 38–50]. The literature screening process and results are illustrated in Fig. 1.

### Basic characteristics of included studies

#### Basic characteristics of CLABSI models

This study included three CLABSI prediction model studies [9, 38, 39]. The outcome measure was consistently defined as CLABSI across all three models. Geographically, one study was conducted in the United States and two in China. All studies employed a retrospective design, with data sourced from electronic health record (EHR) systems. The study populations were focused on specific pediatric subpopulations and did not cover the broader pediatric population. The core modeling method was uniformly LR, with no application of complex algorithms such as machine learning. Missing data were primarily handled using deletion and multiple imputation methods. Detailed information on sample sizes, missing data handling, and predictors is presented in Tables 2 and 3.

**Table 2:** The basic characteristics of the included studies

Author	Year	Country	Participants	Study design	Data Source	Outcome	Missing data	Complication
Laura M [38]	2020	USA	Pediatric emergency department patients	Retrospective study	Medical records	CLABSI	Missing data deletion	CLABSI
Zhang [9]	2025	China	ICU pediatric patients	Retrospective study	EHR	CLABSI	Multiple imputation; Deleted variables with > 20%missing data	
Kuai [39]	2024	China	ICU pediatric patients	Retrospective study	EHR	CLABSI	-	
Jiang [40]	2022	China	Pediatric patients after congenital heart disease surgery	Retrospective study	EHR	CADVT	Deletion of cases with incomplete data	CRT
Xu [41]	2024	China	Pediatric patients with non-tunneled CVC placement	Retrospective study	EHR	CRT	Missing data deletion	
Marquez [42]	2016	USA	ICU pediatric patients	Retrospective study	EHR	CADVT	-	
Li [43]	2021	China	ICU pediatric patients	Retrospective study	EHR	CADVT	-	
Li [19]	2022	China	ICU pediatric patients	Retrospective study	EHR	CADVT	-	
Zhang [44]	2025	China	Pediatric patients with Acute Leukemia (AL)	Retrospective study	EHR	VTE	-	
Zhang [45]	2023	China	Pediatric patients receiving blood purification therapy	Retrospective study	EHR	CRT	Deletion of cases with > 20%missing data	
Fu [46]	2025	China	Pediatric patients with hematologic malignancies	Retrospective study	EHR	PICC-related thrombosis	Deletion of variables with > 20%missing data	
Xie [47]	2024	China	Children using CVC	Retrospective study	EHR	CRT	-	
Fu [48]	2025	China	Children undergoing central venous catheterization	Retrospective&Prospective study	EHR	CRT	Multiple imputation; Deleted variables with > 20%missing data	
Jiang [49]	2025	China	Children with congenital heart disease	Retrospective study	EHR	CRT	Missing data deletion	
Tian [50]	2023	China	Hospitalized children	Retrospective study	EHR	CRT	Multiple imputation	

The ‘Outcome’ presents the outcome measures as originally defined in each included study, the core outcomes have all been verified to align with the definition of CRBSI/CRT in this study, ensuring data consistency

*EHR* Electronic Health Record system

**Table 3:** Model construction methods and performance

Author	Sample Size	Development model method	Verification model method	Model performance			Complication
				AUC(MD/IV/EV)	Calibration method	Specificity/Sensitivity/Accuracy	
Laura M [38]	599	LR	-	0.81	-	98.5%/28.6%/-	CLABSI
Zhang [9]	312	LR	Bootstrap Internal Validation	0.793	Calibration Curve	69.1%/78.6%/-	
Kuai [39]	MD:160; IV:50	LR	IV: Hosmer-Lemeshow (H-L) test + ROC curve; EV: Independent validation cohort	0.812	H-L	MD:0.872/0.904/-; AIV:0.800/0.850/0.84	
Jiang [40]	MD:234; EV:60	LR	IV: H-L test + ROC curve; EV: Independent validation set	MD:0.957;EV:0.922	H-L	MD:96.2%/85.1%/-; EV:85.7%/87.0%/86.67%	CRT
Xu [41]	MD:260; IV:128	LR	IV: H-L test+Calibration Curve + ROC curve	MD:0.857;IV:0.846	Calibration Curve、H-L	MD:88.7%/77.3%/78.5%; IV:86.5%/76.9%/80.5%	
Marquez [42]	175	LR+CART/CHAID	10-fold Cross-Validation	Model 1/2/4:0.80; Model 3:0.75	-	-	
Li [43]	1830	Multimodal Deep Learning Model(LSTM + GRU+FNN)	5-fold Cross-Validation	0.82	Brier Score + Spiegelhalter’s z test	-/-/75%	
Li [19]	3871 patients, 3927 catheters	Fusion Model(Stacking+Blending) based on LR, RF, GBDT	5-fold Cross-Validation	0.82	-	-/-/65%	
Zhang [44]	120	LR + RF	ROC curve, AUC, Delong test	RF:0.860;LR:0.775	-	-	
Zhang [45]	286	LR	IV: H-L test + ROC curve; EV: Independent sample	MD:0.765;EV:0.745	H-L	MD:82.30%/65.40%/-; EV:64.50%/76.90%/-	
Fu [46]	MD:363; IV:155	LR	Bootstrap Internal Validation	MD:0.844;IV:0.794	Calibration Curve, H-L	MD:79.4%/79.1%/-;IV:86.7%/61.6%/-	
Xie [47]	MD:553; IV:116	LR + XGBoost	MD + EV: Time-split validation set	LR: MD:0.872, IV:0.778; XGBoost: IV:0.812	Calibration Curve	-	
Fu [48]	MD:793;IV:340;EV:312	LASSO + LR、RF、ANN、XGBoost	MD + IV+EV	IV:0.884;EV: 0.850	H-L、Calibration Curve	IV:88.9%/76.7%/86.8%; EV:74.2%/84.6%/91.1%	
Jiang [49]	MD:403; IV:100	LR	MD + IV	MD: 0.866;IV༚0.761	H-L、Calibration Curve	MD:85.1%/78.9%/NPV-PPV; IV:75.9%/81.0%/NPV-PPV	
Tian [50]	MD:352;IV:151;EV:85	LR	MD + IV+EV	MD:0.74;IV:0.71;EV:0.76;	H-L、Calibration Curve	MD:51%/86%/79%; IV: 75%/56%/72%; EV: 88%/54%/71%	

*MD* Model Development Group, *IV* Internal Verification Group, *EV* External Verification Group, *LR* Logistic Regression, *RF* Random Forest, *XGBoost* eXtreme Gradient Boosting, *GBDT* Gradient Boosting Decision Tree, *LASSO* Least Absolute Shrinkage and Selection Operator, *CART* Classification and Regression Trees, *CHAID* Chi-squared Automatic Interaction Detector, *LSTM* Long Short-Term Memory, *ANN* Artificial Neural Network, *GRU* Gated Recurrent Unit, *FNN* Feedforward Neural Network, *AUC* Area Under the ROC Curve, *H-L test* Hosmer-Lemeshow test *ROC* Receiver Operating Characteristic, -: Not reported

#### Basic characteristics of CRT prediction models

A total of 12 studies on CRT prediction models were included [19, 40–50]. Geographically, the studies were highly concentrated in China (11 studies), with only one from the United States. The study designs were predominantly retrospective (11 studies), with only one employing a mixed retrospective and prospective design. All studies utilized data derived from EHR. The study populations covered various pediatric subgroups, including post-cardiac surgery for congenital heart disease, pediatric ICU, and acute leukemia, with a wide range of sample sizes. Modeling methods were relatively diverse, encompassing not only LR but also algorithms such as Random Forest, XGBoost, and deep learning. Handling of missing data mainly involved deleting cases/variables with high missing rates and multiple imputation. Notably, nearly half of the studies did not explicitly report their handling strategies. Specific characteristics are detailed in Tables 2 and 3.

#### Overall characteristics of the included prediction models

Among the 15 included studies, 14 (87.5%) were published between 2021 and 2025, while only 1 were published before 2021 (in 2016). This indicates a significant increase in research attention on risk prediction models for pediatric CVC-related complications in recent years. Notably, the application of machine learning algorithms in model construction has been gradually increasing, with 5 models employing machine learning or hybrid algorithms, all published between 2022 and 2025.

### Construction methods and predictive performance of included models

#### Development and performance of CLABSI prediction models

All three CLABSI prediction models employed LR as the sole modeling method [9, 38, 39]. In terms of discriminative ability, the Area Under the Curve (AUC) for model derivation ranged from 0.793 to 0.812. Validation was insufficient: only one study conducted both internal and external validation [39], one study performed only internal validation [9], and one study did not perform any validation [38]. The application of calibration methods was limited; only two studies reported calibration-related metrics (calibration plots, Hosmer-Lemeshow (H-L) test) [9, 39], while one study did not report any calibration information [38]. The models were presented in various forms, including scoring scales, nomograms, and regression equations. The core predictors were primarily centered on three categories: catheter characteristics, nursing procedures, and the clinical status of pediatric patients. Their predictive performance solely reflects the ability to discriminate CLABSI. Detailed performance metrics are presented in Tables 3 and 4.

**Table 4:** Model Report of the Malnutrition Risk Prediction Model

Author	Candidate Variables	Model presentation	The number of predictors	Final predictors	Complication
Laura M [38]	26	Score Chart	12	Age≤5years; Black race༛Use of total parenteral nutrition༛Tunneled central venous catheter༛Double lumen catheter༛Absence of other bacterial infection༛Absence of viral upper respiratory infection symptoms༛Diarrhea༛Emergency department temperature > = 39.5 degrees Celsius༛Fever prior to presentation༛Neutropenia༛Spring/summer season.	CLABSI
Zhang [9]	9	Nomogram+Regression Equation	4	Number of dressing changes(Protective factor), Catheter indwelling time(Risk factor), Heparin lock(Protective factor), Repeated catheterization(Risk factor).	
Kuai [39]	16	Regression Equation	6	Catheter-inserting nurse’s years of experience, Number of puncture attempts, Catheter indwelling time, Multi-lumen central venous catheter, Serum albumin < 35 g/L, Untimely dressing change.	
Jiang [40]	23	Regression Equation+Nomogram	6	CVC indwelling duration, D-dimer level, Fibrinogen level, Days of sedation, Pediatric Critical Illness Score, Number of types of vasoconstrictors used.	CRT
Xu [41]	16	Nomogram	5	Age (in months), Use of corticosteroids, Parenteral nutrition support, CVC site (Internal jugular vein), Multi-lumen catheter (vs. single-lumen).	
Marquez [42]	10	Decision Tree(CART)	4	Age, Recent surgery, CVC in subclavian vein, Blood transfusion.	
Li [43]	143 static + 56 dynamic	Neural Network Architecture Diagram	-	Demographic data, Clinical diagnosis, Laboratory indicators, Vital signs, Medication use, etc.	
Li [19]	478	Feature Importance Ranking Table	11	SBE, TT difference, Dehydrating agents, HCO₃, PaO₂, D-dimer, Basophils, Thrombin time (TT), Lymphocytes (LY%), pH, Surgery history.	
Zhang [44]	12	Score Chart	6	Puncture site, Number of puncture attempts, Catheter indwelling time, Catheter-related complications, Use of anticoagulants, D-dimer level.	
Zhang [45]	22	Nomogram	6	Age, Line clotting, Catheter dysfunction, Catheter indwelling time, Subtherapeutic anticoagulation, Pediatric Critical Illness Score(Protective factor).	
Fu [46]	54	Nomogram	6	Leukemia, Number of catheters ≥ 2, History of catheterization, TPN, Post-catheterization D-dimer, Post-catheterization fibrinogen.	
Xie [47]	8	Nomogram	6	Catheterization duration, Sex, Disease type(e.g., Congenital heart disease), Postoperative status, Femoral vein catheterization.	
Fu [48]	60	Nomogram	11	History of thrombosis, Leukemia, Number of catheters, History of catheterization, Chemotherapy, Parenteral nutrition, Mechanical prophylaxis, Dialysis, Hyperosmolar fluids, Anticoagulants, Post-catheterization D-dimer.	
Jiang [49]	26	Dynamic Online Nomogram	5	Catheter indwelling time, ICU length of stay, Duration of sedation, Fibrinogen ≥ 4 g/L, Platelets ≥ 400 × 10⁹/L.	
Tian [50]	30	Nomogram	7	Blind insertion catheterization, Abnormal liver function, Central Line-Associated Bloodstream Infection(CLABSI), Infection, Number of catheterizations, Leukemia, Bed rest > 72 h.	

#### Development and performance of CRT prediction models

The 12 CRT prediction models exhibited diversity in modeling approaches: six used LR alone [40, 41, 45, 48–50]; five combined LR with machine learning algorithms (such as Random Forest, XGBoost) [42–44, 47, 48]; and one employed a multimodal deep learning model [43]. The modeling AUC ranged from 0.74 to 0.957, with the model for post-operative CRT in congenital heart disease demonstrating the best performance (development cohort AUC = 0.957, external validation AUC = 0.922) [40]. Validation coverage was superior to that of the CLABSI models, with 4 studies conducting both internal and external validation [40, 45, 48, 50], and 3 studies performing only internal validation [41, 46, 49]. Calibration methods were more extensively applied, often combining the H-L test with calibration curves. The models were primarily presented as nomograms. Core predictors included laboratory indicators (D-dimer, fibrinogen), underlying diseases, and catheter-related factors. Specific performance indicators are detailed in Tables 3 and 4.

### Results of bias risk and applicability assessment

Table 5 summarizes the results of the risk of bias and applicability assessments for the models from all included studies. In the domain of study participants, 14 studies had a high risk of bias [9, 19, 38–47, 49, 50], while one study had a low risk of bias [48].

**Table 5:** PROBAST results of the included studies

Author	ROB					Applicability			
	Predictors	Predictors	Outcome	Analysis	Overall ROB	Predictors	Predictors	Outcome	Overall Applicability
Laura M [38]	②	②	①	②	②	①	①	①	①
Zhang [9]	②	②	①	①	②	①	①	①	①
Kuai [39]	②	②	①	②	②	①	①	①	①
Jiang [40]	②	②	①	②	②	①	①	①	①
Xu [41]	②	②	①	②	②	①	①	①	①
Marquez [42]	②	②	②	②	②	①	①	①	①
Li [43]	②	①	②	②	②	①	①	①	①
Li [19]	②	①	②	②	②	①	①	①	①
Zhang [44]	②	①	②	②	②	①	①	①	①
Zhang [45]	②	①	②	①	②	①	①	①	①
Fu [46]	②	②	①	②	②	①	①	①	①
Xie [47]	②	③	②	①	②	①	①	①	①
Fu [48]	①	①	①	①	①	①	①	①	①
Jiang [49]	②	③	①	①	②	①	①	①	①
Tian [50]	②	②	②	①	②	①	①	①	①

*PROBAST* Prediction model Risk Of Bias Assessment Tool, *ROB * risk of bias

①indicates low ROB/low concern regarding applicability; ②indicates high ROB/high concern regarding application; ③indicates unclear ROB/unclear concern regarding applicability

#### Risk of bias characteristics by model type

##### Prediction models for CLABSI

All CLABSI prediction model studies were assessed as having a high risk of bias. This primarily stemmed from selection bias due to single-center retrospective designs, coupled with issues such as highly subjective predictors (e.g., adherence to standardized nursing practices) and potential missing data for infection diagnoses (e.g., blood culture results). In the statistical analysis domain, there was a risk of overfitting, and external validation was weak, with only one study conducting external validation and not separately reporting the AUC value [39].

##### Prediction models for CRT

Among the 12 studies, only one demonstrated a low risk of bias [48], while the others were rated as having a high or unclear risk of bias. In addition to common issues such as single-center, retrospective designs, the risk of bias in the outcomes domain was particularly prominent. This was due to the potential under-detection of outcomes, as some asymptomatic thrombi may not have been identified by ultrasound screening. The statistical analysis domain also faced problems, including imbalances between sample size and the number of variables, as well as improper handling of missing data.

#### Results of applicability assessment

The applicability assessment indicated that all models demonstrated high alignment with the objectives of this study across three dimensions: study participants (pediatric patients with CVCs), predictors (routine clinical indicators), and outcomes (CLABSI/CRT). Overall applicability was satisfactory. However, it is noteworthy that outcome measurements in some models were flawed (e.g., inconsistent diagnostic criteria for CRT, missing blood culture data related to infections), which limits the practical significance of model validation. Even when the applicability assessment was rated as ‘low concern,’ the reliability of the results requires cautious interpretation.

## Discussion

### Model performance and overall characteristics

The models included in this systematic review demonstrate some potential in terms of discrimination. However, limited by a high risk of bias, their actual clinical value requires comprehensive evaluation using multi-dimensional metrics rather than relying solely on AUC values. Regarding core discriminative ability, the pooled AUC range for CLABSI prediction models was 0.793–0.812, and for CRT prediction models, it was 0.74–0.957, indicating that most models can reasonably distinguish between high-risk and low-risk pediatric patients. Among them, the CRT model for post-operative congenital heart disease performed the best in both the development cohort (AUC = 0.957) and the external validation cohort (AUC = 0.922) [40], showing outstanding discriminative ability in a specific pediatric subgroup. However, due to limitations such as single-center design and restricted validation, careful consideration is warranted when considering its clinical generalization.

#### Comprehensive assessment of model performance

Relying solely on AUC to evaluate model performance has clear limitations; assessment must be further refined by considering calibration effectiveness, validation completeness, and sample size appropriateness: ① Insufficient Reporting and Application of Calibration Metrics: Calibration is a critical prerequisite for clinical decision-making; however, the included studies paid insufficient attention to calibration. Among the 15 studies, only 8 used the H-L test and only 7 employed calibration curves for evaluation. Some studies simply deemed calibration acceptable based solely on the H-L test (*P* > 0.05), lacking visual analysis via calibration curves. This may result in models with “strong discriminative ability but poor prediction accuracy.” ② Insufficient validation completeness and weak external validation: In this review, only 5 studies conducted external validation, and some of these validations had performance limitations (e.g., overly small sample sizes, failure to report key metrics). The remaining 10 studies only completed model development or performed only internal validation, potentially leading to overestimated model performance. ③ Imbalance between sample size and number of variables, with high risk of overfitting: Most models suffered from the issue of “small sample size with many candidate variables,” resulting in an insufficient number of Events Per Variable (EPV). This significantly increases the risk of overfitting.

#### Geographical distribution and modeling method characteristics of the models

The two types of models exhibit differences in geographical distribution and modeling approaches. Regarding geographical distribution, the CLABSI models were developed using a limited dataset and lack representativeness, whereas the CRT models are highly concentrated in China (11 out of 12 studies). This disparity reflects differences in healthcare systems and research priorities across countries; however, due to language restrictions in the search strategy, the global representativeness of the models remains questionable. In terms of modeling approaches, all CLABSI models employed LR as the sole modeling method, whereas the CRT models adopted more diverse approaches, including LR, machine learning, and deep learning algorithms. This is closely related to the greater difficulty in developing CLABSI models, the relatively smaller sample sizes available, whereas CRT models benefit from a wider range of sample sizes and better data accessibility.

#### Analysis of differences in complication types

##### Reasons for differences in research focus

The number of CRT prediction models (12 studies) far exceeds that of CLABSI prediction models (3 studies), most likely for two reasons. First, the incidence of CRT in children (14%–28%) [19, 20] is significantly higher than that of CLABSI, creating a more urgent clinical demand. Second, the diagnosis of CRT relies on imaging examinations such as Doppler ultrasound and CT, which provide objective and traceable results, making data extraction more feasible in retrospective studies. In contrast, the diagnosis of CLABSI depends on blood culture results and the exclusion of other infection sources [51]. Data in this area are more prone to missingness or inconsistencies in diagnostic criteria, which constrains the conduct of such research.

##### Differences in predictors

The predictors of the two types of models demonstrate distinct domain specificity: predictors for CLABSI focus on the quality of nursing practices (such as the number of catheter insertion attempts and timely dressing changes) [39] and catheter structural characteristics (such as multi-lumen catheters and tunneled CVCs) [38]. In contrast, predictors for CRT primarily consist of laboratory indicators (e.g., D-dimer, fibrinogen) [40, 46], underlying diseases (e.g., leukemia, congenital heart disease) [44, 49], and treatment-related factors (e.g., glucocorticoid use, parenteral nutrition) [41]. This core difference is directly related to the distinct pathophysiological mechanisms of the two types of complications.

### Methodological quality and risk of bias

According to the PROBAST assessment results, only one of the included studies was rated as having a low risk of bias [48], while all others were judged to have a high or unclear risk of bias. All included studies relied on EHR data, which introduces inherent methodological limitations: ① Diagnostic data for CLABSI (such as blood culture results and infection source investigation) may be subject to coding errors or missingness, leading to outcome misclassification; ② Certain predictors (such as dressing change timing and compliance with standardized catheter insertion protocols) may be incompletely recorded in EHRs or are highly subjective, affecting the accuracy of model development. ③ Issues such as an imbalance between sample size and the number of candidate variables, improper handling of missing data, and weak external validation are widespread. These methodological shortcomings—including single-center retrospective design, limitations in data extraction, and insufficient validation and calibration—collectively contribute to the high risk of bias in the existing models, which is the core reason for their limited reliability.

### Clinical implications and translational challenges

An ideal risk prediction model can empower precision medicine by identifying high-risk pediatric patients, enabling optimized allocation of medical resources and proactive, individualized interventions [52]. However, translating existing models into clinical practice still faces significant barriers. First, high risk of bias and insufficient validation undermine the foundational reliability of these models. Clinicians may find it difficult to trust a predictive tool that could potentially fail in their own clinical setting. Second, the usability and integration of the models are extremely limited. Most models are presented as static nomograms or regression equations, lacking integration into electronic health record systems. This prevents real-time, automated risk calculation and greatly restricts their clinical applicability. This finding is highly consistent with a systematic review of Ventilator-Associated Pneumonia (VAP) prediction models [53], who noted that most models exhibit low technical readiness and a lack of workflow integration. Given that current models are predominantly single-center retrospective studies with a high risk of bias and inadequate external validation, they currently hold only exploratory value. They have not yet met the reliability standards required for clinical application and should not be used as standalone tools for clinical decision-making.

### Comparison with and insights from adult literature

Comparing our findings with research on prediction models in adult internal medicine provides valuable insights. In a systematic review of VAP prediction models [53], machine learning models demonstrated excellent discriminative performance (pooled AUROC 0.88), but they were similarly plagued by high risk of bias and low technical readiness. That review highlighted the value of dynamic prediction models and feature importance analysis, which aligns with our own findings. For pediatric CVC complications, future models should also strive to utilize temporal data for dynamic risk prediction updates and clarify the key factors driving predictions to enhance clinicians’ understanding and trust. In a systematic review on the association between infection and VTE [54], researchers confirmed through meta-analysis that infection is an independent risk factor for VTE (OR 2.69–5.80). This provides pathophysiological support for our findings: a CVC itself represents an endothelial injury, which, if combined with CLABSI, may significantly increase thrombotic risk through the mechanism of immunothrombosis. This implies that infection-related indicators should be considered an important category of predictors when developing pediatric CVC thrombosis prediction models. However, that review also pointed out that classic VTE risk assessment tools such as Caprini and Padua assign low weight to or even disregard infection [55, 56]. This cautions us that future development of pediatric-specific models must be based on pediatric evidence, thoroughly evaluate, and integrate such critical risk factors. 

### Implications for future research and practice

Future development of prediction models should focus on: ① Standardization of Diagnostic Methods and Data Enhancement: Further standardize the clinical diagnostic criteria for CLABSI based on CDC guidelines, clarifying the requirements for recording blood culture results and infection symptoms to reduce outcome misclassification bias in retrospective studies. For CRT, adopt a combined diagnostic approach using ultrasound and laboratory indicators (e.g., D-dimer). Simultaneously, enhance EHR data recording by standardizing subjective indicators such as the standardization of nursing procedures and healthcare-associated infection control measures, thereby providing high-quality data for the development of CLABSI models. ② Refinement of baseline data and consistent variable definitions: Model development should involve the collection of comprehensive baseline data, with clear operational definitions of predictors to minimize bias arising from subjectivity. ③ Stratification of high-risk populations: Specific models should be developed separately for high-risk subgroups such as neonates and children with hematologic malignancies to enhance model relevance. ④ Optimization of study design and validation processes: The quality of models should be improved through multicenter, prospective cohort designs, and external validation should be strengthened to ensure generalizability. ⑤ Promotion of clinical integration: Models should be embedded into electronic health record systems to enable real-time risk calculation and seamless integration with clinical workflows. Future efforts must precisely address the clinical characteristics of both types of complications to ultimately achieve the pediatric care objective of reducing complication rates.

### Strengths and limitations of the study

This study systematically reveals the methodological flaws and clinical limitations of existing risk prediction models for pediatric CVC-related complications, providing critical insights for future model optimization. The PROBAST tool was employed to scientifically assess the quality of the prediction models, ensuring methodological rigor. However, the study also has several limitations. First, the systematic search was limited to Chinese and English literature. Given that countries like Italy and Germany have active research communities in pediatric intravenous therapy, relevant models from these regions may have been missed. Future systematic reviews could expand the search to include studies in additional languages to enhance the global representativeness of the findings. Second, some subjectivity is inherent in the quality assessment process, a common bias in systematic reviews. To minimize this, two independent reviewers used the PROBAST tool for assessment, and any disagreements were resolved through discussion or third-party arbitration. Third, the analysis of predictors in this study was descriptive; a lack of in-depth quantitative analysis may render the evaluation results relatively limited. Finally, only three CLABSI-related models were included. Although a consistent outcome measure was achieved, the small sample size may result in an incomplete summary of the characteristics of CLABSI models. Future efforts should focus on conducting more large-sample, multicenter studies on CLABSI models to enrich the evidence base for systematic reviews.

## Conclusion

This systematic review evaluated 15 risk prediction models for pediatric CVC-related complications (3 for CLABSI and 12 for CRT). The results indicate that while the models show some potential in terms of discrimination (AUC range 0.74–0.957), they are generally limited by issues such as a high risk of bias, single-center design, and insufficient validation. Currently, these models can only serve as reference points for clinical risk screening. The findings highlight the need for future multicenter prospective studies to standardize diagnostic criteria and data collection procedures, optimize model development and validation methodologies, focus on model construction for high-risk populations, and promote the integration of models with electronic medical record systems. These steps are essential to enhance the clinical applicability of the models and ultimately achieve precise prevention and control of CVC-related complications in children.

## Supplementary Information

Supplementary Material 1.

## Data Availability

All data generated or analysed during this study are included in this published article [and its supplementary information files].

## Abbreviations

CVC: Central Venous Catheters
CLABSI: Central Line-Associated Bloodstream Infection
CRT: Catheter-Related Thrombosis
CNKI: China National Knowledge Infrastructure
PRISMA: Preferred Reporting Items for Systematic Review and Meta-Analysis
CHARMS: Checklist for critical appraisal and data extraction for systematic reviews of prediction modelling studies
PROBAST: Prediction Model Risk of Bias Assessment Tool
EPV: Events Per Variable
PICU: Pediatric Intensive Care Unit
CADVT: Central Access Device-Related Thrombosis / Central Venous Access Device-Associated Thrombosis
VTE: Venous Thromboembolism
PICC: Peripherally Inserted Central Catheter
MD: Model Development
IV: Internal Validation
EV: External Validation
LR: Logistic Regression
RF: Random Forest
GBDT: Gradient Boosting Decision Tree
LASSO: Least Absolute Shrinkage and Selection Operator
ANN: Artificial Neural Network
XGBoost: Extreme Gradient Boosting
CART: Classification and Regression Trees
CHAID: Chi-squared Automatic Interaction Detector
LSTM: Long Short-Term Memory
GRU: Gated Recurrent Unit
FNN: Feedforward Neural Network
AUC: Area Under the Curve
H-L test: Hosmer-Lemeshow test
ROC: Receiver Operating Characteristic
