# Supplementary material for: Risk prediction models for central venous catheter-related complications in children: a systematic review
Source: BMC Pediatr. 2026 Apr 27;26:558. doi: 10.1186/s12887-026-06919-y (PMC13255339; doi:10.1186/s12887-026-06919-y)
Supplement: Supplementary file 1 — Supplementary Material 1. [file 12887_2026_6919_MOESM1_ESM.docx]

**Search strategy**

**中文检索词:**

**#1 幼儿 OR 儿童 OR 儿科**

**#2 中心静脉导管 OR** 经外周静脉置入中心静脉导管 **OR 输液港 OR CVC OR PICC OR PORT**

**#3 并发症 OR 感染 OR 导管相关血流感染 OR CRBSI OR CLABSI OR 血栓 OR 导管相关性血栓**

**#4 风险预测 OR 预测模型 OR 风险评分 OR 列线图 OR 预测因子 OR 预测因素 OR 机器学习**

****Pubmed:****

**#1** Child[MeSH Terms] OR Children[MeSH Terms] OR Pediatric[MeSH Terms]

#2 central venous catheters[MeSH Terms] OR peripherally inserted central cather[MeSH Terms] OR central venous catheters[MeSH Terms] OR PICC[MeSH Terms] OR CVC[MeSH Terms] OR PORT[MeSH Terms]

#3 Complication*[Title/Abstract] OR Adverse Effects[Title/Abstract] OR “Blood-Borne Infect*”[Title/Abstract] OR “Catheter-Related Infect*”[Title/Abstract] OR CRBSI[Title/Abstract] OR CLABSI[Title/Abstract] OR “Bloodstream Infect*”[Title/Abstract] OR Blood Poison*[Title/Abstract] OR Venous Thrombosis[Title/Abstract] OR “Catheter-Related Thrombosis”[Title/Abstract] OR “Blood Clot*”[Title/Abstract] Or CRT[Title/Abstract]

#4 Risk Predict*[Title/Abstract] OR Risk Score[Title/Abstract] OR Risk Assess*[Title/Abstract] OR Score[Title/Abstract] OR Risk Model[Title/Abstract] OR Nomogram* Model[Title/Abstract] OR Score Development[Title/Abstract] OR Risk Calculator[Title/Abstract] OR Machine Learning[Title/Abstract]

#5 #1 AND #2 AND #3 AND #4
